# Integrating Full‐Color 2D Optical Waveguide and Heterojunction Engineering in Halide Microsheets for Multichannel Photonic Logical Gates

**DOI:** 10.1002/advs.202310262

**Published:** 2024-02-29

**Authors:** Chang Xing, Bo Zhou, Dongpeng Yan, Wei‐Hai Fang

**Affiliations:** ^1^ Key Laboratory of Theoretical and Computational Photochemistry, Ministry of Education, College of Chemistry Beijing Normal University Beijing 100875 P. R. China

**Keywords:** heterojunction, metal‐organic halides, optical waveguide, room temperature phosphorescence, space‐time‐color resolution

## Abstract

Ensuring information security has emerged as a paramount concern in contemporary human society. Substantial advancements in this regard can be achieved by leveraging photonic signals as the primary information carriers, utilizing photonic logical gates capable of wavelength tunability across various time and spatial domains. However, the challenge remains in the rational design of materials possessing space‐time‐color multiple‐resolution capabilities. In this work, a facile approach is proposed for crafting metal‐organic halides (MOHs) that offer space‐time‐color resolution. These MOHs integrate time‐resolved room temperature phosphorescence and color‐resolved excitation wavelength dependencies with both space‐resolved ex situ optical waveguides and in situ heterojunctions. Capitalizing on these multifaceted properties, MOHs‐based two‐dimensional (2D) optical waveguides and heterojunctions exhibit the ability to tune full‐color emissions across the spectra from blue to red, operating within different spatial and temporal scales. Therefore, this work introduces an effective methodology for engineering space‐time‐color resolved MOH microstructures, holding significant promise for the development of high‐density photonic logical devices.

## Graphical abstract

Luminescent metal‐organic halide (MOH) microcrystals (L‐CdCl_2_ and L‐CdI_2_) could serve as 2D full‐color optical waveguides that transmit photon signals with ex situ space‐time‐color triple resolution characteristics. Moreover, the dynamic manipulation of heterojunctions (MOH‐Cl@I and MOH‐I@Cl) can be achieved, which exhibit interesting in situ space‐time‐color triple resolution features to further develop advanced photonic logical gates.

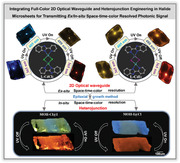

## 1 Introduction

**Figure 1:**
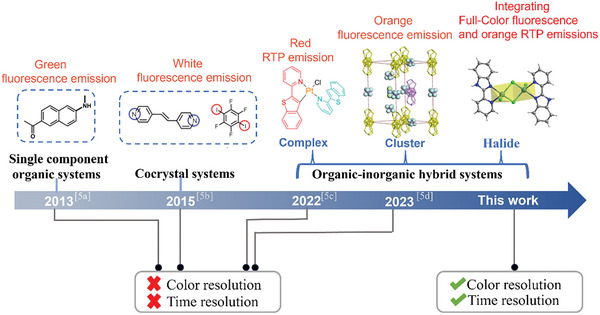
A roadmap illustrating the evolution of 2D active optical waveguides.

With the rapid development of science and technology, ensuring information security in communication processes has become a pivotal factor for the steady progress of modern society. Given the limitations of traditional electronic devices, including slow computing speeds, high energy consumption, and the risk of information leakage, it has become evident that constructing photonic systems offers a viable alternative to mitigate these drawbacks associated with electronics.^[^ ^1^ ^]^ In this context, photonic logical gates, which serve as important components in photonic circuits, have garnered significant attention due to their substantial storage capacity and high‐speed data transmission capabilities.^[^ ^2^ ^]^ Optical waveguides, responsible for transporting photons within photonic logical gates from their source to the destination, represent a typical medium for ex situ photon signal propagation. Unlike conventional passive optical waveguides, such as glass fibers, active optical waveguides possess the unique ability to absorb input light driven by additional energy and propagate the resulting irradiated light, thereby facilitating multi‐mode photonic computation via their highly tunable photophysical properties.^[^ ^3^ ^]^ In contrast to one‐dimensional (1D) optical waveguides, two‐dimensional (2D) systems, which confine and transport photons in a plane alone in different directions, have emerged as an ideal choice for constructing photonic logical gates.^[^ ^4^ ^]^ To date, 2D active optical waveguides remain a rare development, limited primarily to single‐component purely organic and cocrystal systems (**Figure** **1**).^[^ ^4^, ^5^ ^]^

Several strategies, including isotropic propagation, anisotropic propagation, and refractive‐index perturbation, have been explored to enhance photon utilization in 2D optical waveguides.^[^ ^5a^ ^]^ Nevertheless, the progress in developing 2D active optical waveguides has been impeded primarily by challenges in material systems and design methodologies. Quite recently, researchers have endeavored to create new optical waveguide media based on organic–inorganic hybrids by leveraging the strengths of individual components. For example, Zhu et al. have described the active waveguide performance of ligand‐protected atomically precise metal nanoclusters, which exhibit lower optical loss coefficients compared to most purely organic materials.^[^ ^6^ ^]^ Furthermore, we hypothesize that the integration of color and time resolution dimensions into spatially‐resolved 2D optical waveguides could significantly extend the capabilities of multi‐mode photonic logical gates, potentially breaking through the current developmental bottleneck. Unfortunately, such systems remain largely unexplored due to the absence of effective designs that combine full‐color output with long‐lived photoemission.

Theoretically, excitation‐dependent luminescence has the potential to generate multi‐color output spanning from blue to red irradiation scope by varying the excitation wavelength.^[^ ^7^ ^]^ Moreover, it is noteworthy that long‐lived room temperature phosphorescence (RTP) typically ranges from microseconds to seconds, as a result of spin‐forbidden transitions between singlet and triplet excited states, distinguishing it from traditional fluorescence emission.^[^ ^8^ ^]^ Therefore, the ideal 2D optical waveguide with color‐time‐space resolved characteristics is expected to be realized by incorporating excitation‐dependent RTP emission properties.

Different from ex situ spatially‐resolved 2D optical waveguides, crystalline heterostructures that integrate two or more units into a single material offer in situ spatial resolution, enabling the detection of photon signals at the excited position. These heterostructures play a significant role in photonic logical gates by leveraging their synergistic effects on various components.^[^ ^9^ ^]^ Thus far, strategies for fabricating heterojunctions with domain‐controlled colors have been developed, such as core–shell or striped heterojunctions achieved through liquid‐phase anisotropic epitaxial growth.^[^ ^10^ ^]^ However, most of the generated photonic signals in heterojunctions originate from fluorescence with short‐lived nanosecond lifetimes.^[^ ^11^ ^]^ To enhance the level of information encryption in photonic logical gates, it is imperative to develop new heterojunctions with time‐resolved RTP performance.

Metal‐organic halides (MOHs), assembled from metal halides and organic cations, are considered promising candidates for constructing dynamic space‐time‐color resolved photonic logical gates for several compelling reasons: 1) The highly ordered structure and dense stacking resulting from the combination of inorganic and organic components are conducive to generating space‐resolved optical waveguide performance.^[^ ^12^ ^]^ 2) Halogen ions with similar coordination capabilities provide the opportunity to construct multicolor heterojunctions by adjusting the type of halogen ions.^[^ ^13^ ^]^ 3) MOHs have the potential to emit time‐resolved RTP due to the presence of heteroatoms (e.g., N, O, S, or P) and central metal cations with *d* ^10^ configurations (Cd^2+^ or Zn^2+^), which promote spin‐orbit coupling (SOC) and enhance intersystem crossing (ISC);^[^ ^2^, ^14^ ^]^ 4) The abundance of charge transfer paths, such as metal‐to‐ligand charge transfer (MLCT), ligand‐to‐metal charge transfer (LMCT), ligand‐to‐ligand charge transfer (LLCT), and locally excited (LE) states, enables MOHs to exhibit excitation‐dependent multicolor emission.^[^ ^15^ ^]^ 5) The MOHs could often exhibit excellent thermal and solvent stability, which can be used as building blocks for the preparation of heterojunctions.^[^ ^2b^ ^]^

**Figure 2:**
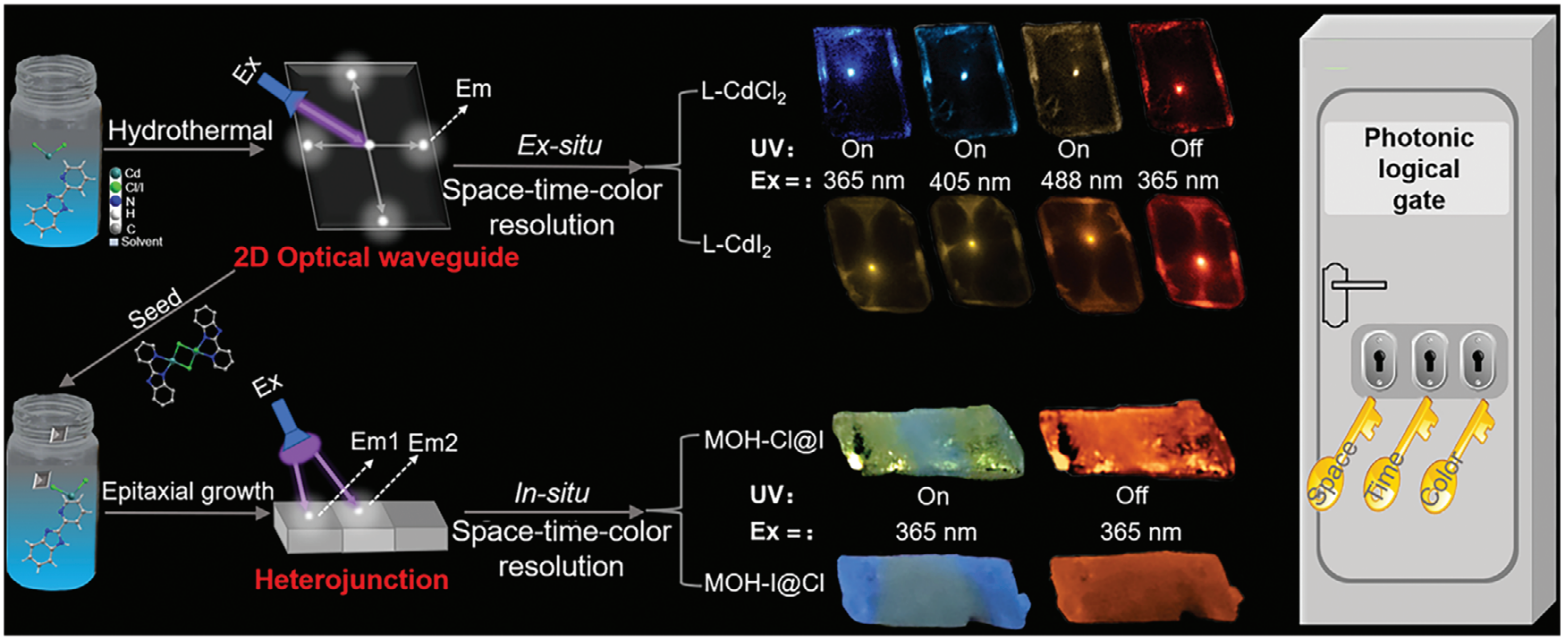
Schematic representation and applications of 2D optical waveguides with ex situ space‐time‐color triple resolution and crystalline heterojunctions with in situ space‐time‐color triple resolution based on MOHs microstructures for enhanced photonic logical gates.

Herein, we have designed and synthesized isostructural 2D color‐tunable MOH microcrystals (L‐CdCl_2_ and L‐CdI_2_) with time‐resolved RTP properties. These microcrystals rarely exhibit both ex situ space‐resolved 2D optical waveguides and in situ heterojunction structures (**Figure** **2**). Under focused UV irradiation, the brighter blue and yellow photoluminescence (PL) is observed at the four edges compared to the body of microcrystals for L‐CdCl_2_ and L‐CdI_2_, respectively, representing typical 2D optical waveguide behavior with spatial resolution. By varying the excitation wavelength, fluorescence and RTP colors can be adjusted across nearly the entire visible spectra due to the coexistence of multiple excited states. Consequently, these materials demonstrate the capability of ex situ space‐time‐color triple resolution in 2D optical waveguides, showcasing strong photonic memory capacity through the multichannel transmission of tunable photon signals. Furthermore, we have successfully manipulated heterojunctions (MOH‐Cl@I and MOH‐I@Cl) in the isostructural MOHs using a heteroepitaxial growth method. These heterojunctions exhibit intriguing in situ space‐time‐color triple‐resolution features, paving the way for the development of advanced photonic logical gates. Therefore, this work not only realizes full‐color 2D optical waveguides and heterojunction engineering in MOH microcrystals but also paves an effective way for achieving photonic logical gates with ex situ/in situ space‐time‐color resolution, promising high‐level information storage and security.

## 2 Results and Discussion

2D microcrystals of L‐CdCl_2_ and L‐CdI_2_ can be readily synthesized via a hydrothermal synthesis process. Single‐crystal X‐ray diffraction analysis revealed that both L‐CdCl_2_ and L‐CdI_2_ crystallize in a triclinic crystal system with the *P‐1* space group (Table S1, Supporting Information). In these crystal structures, two organic ligands are connected by two CdX_2_ (X = Cl or I) units, whereby each Cd^2+^ ion adopts a distorted tetrahedral geometry that is five‐coordinated with two nitrogen atoms from the organic ligand and three chlorine ions (Figures S1,S2, Supporting Information). The Cd─N bond lengths range from 2.29 to 2.35 Å, while the Cd‐X bond lengths vary from 2.24 to 2.58 Å. The bond angles around the Cd^2+^ center fall within the range of 85.49° to 115.89°. These independent 0D structures are connected through C─H···X interactions, spanning in the scope of 2.41 to 2.97 Å for L‐CdCl_2_ and 2.82 to 3.14 Å for L‐CdI_2_. Moreover, they exhibit π–π interactions at distances of 3.35 and 3.78 Å, with corresponding dihedral angles of 3° and 0°, respectively (Table S2, Supporting Information). These robust molecular interactions result in dense stacking in both MOHs. Fourier transform infrared (FT‐IR) spectra further confirm the structural similarity between L‐CdCl_2_ and L‐CdI_2_ (Figure S3, Supporting Information). Additionally, when compared to the pristine organic ligand (2‐PyBIM), both MOHs display a broad absorption band spanning from 400 to 700 nm, implying the presence of a charge transfer process (Figure S4, Supporting Information). Powder X‐ray diffraction (PXRD) results confirm that both MOHs are single‐phase (Figure S5, Supporting Information). Thermal gravimetric analysis (TGA) affirms the high thermal stability of these MOHs, with decomposition temperatures at approximately 370 °C for L‐CdCl_2_ and 360 °C for L‐CdI_2_ (Figure S6, Supporting Information).

**Figure 3:**
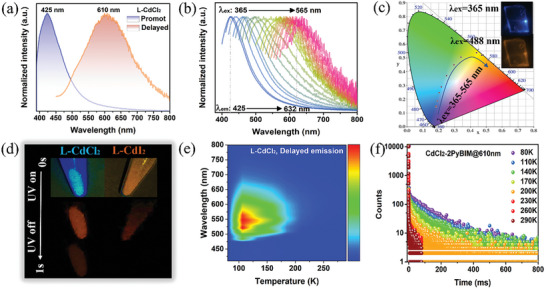
a) Prompt and delayed emission spectra of L‐CdCl_2_; b) Excitation‐dependent prompt emission spectra; c) CIE coordinate diagram of L‐CdCl_2_ as a function of the excitation wavelengths; d) Fluorescence and afterglow photographs; e) Temperature‐dependent delayed spectra and f) time‐resolved delayed decay at different temperatures of L‐CdCl_2_.

To explore the photophysical properties of these MOH materials, PL spectra were conducted under both prompt and delayed (acquired after 1 ms of excitation) modes. Upon UV light irradiation at the optimized wavelength (Figure S7, Supporting Information), the prompt emission spectra of L‐CdCl_2_ and L‐CdI_2_ are illustrated in **Figures** **3**a and S8, Supporting Information, which show that the primary emission bands are located at 425 and 560 nm, respectively. Their corresponding photoluminescence quantum yield (PLQY) values are calculated as 11% and 6.11% (Figure S9, Supporting Information). Decay curves demonstrate identical fluorescence lifetimes of 1.1 ns for both L‐CdCl_2_ and L‐CdI_2_, indicating similar radiative transition behaviors of their singlet excitons (Figure S10, Supporting Information). Notably, both MOH systems exhibit highly color‐tunable photoemission under varying light irradiation. As shown in Figure 3b, the emission peak of L‐CdCl_2_ is gradually red‐shifted from 425 to 632 nm as the excitation wavelength increases from 365 to 565 nm. This shift corresponds to a change in fluorescence color from blue (CIE: 0.183, 0.144) to orange‐red (CIE: 0.438, 0.538) (Figure 3c). L‐CdI_2_ shows a similar excitation‐dependent PL performance, as demonstrated in Figures S11,S12, Supporting Information. An increase in excitation wavelength from 400 to 600 nm induces a redshift in the emission band from 560 (CIE: 0.364, 0.412) to 660 nm (CIE: 0.534, 0.463), confirming the full‐color visible light‐emitting properties of these MOHs.

Furthermore, the prominent emissions observed in the delayed spectra of both L‐CdCl_2_ and L‐CdI_2_ are centered at 610 nm, aligning with the orange‐red afterglow perceived by the naked eye (Figure 3a,d and Figures S8,S13, Supporting Information). Concurrently, L‐CdCl_2_ and L‐CdI_2_ display excitation‐dependent emission properties in the delayed mode (Figure S14, Supporting Information). Significantly, decay curves depict that the afterglow emissions have extended lifetimes measured in milliseconds (Figure S15 and Table S3, Supporting Information), illustrating their time‐resolved afterglow behavior. To gain deeper insights into the nature of the room‐temperature afterglow emission of MOHs, we systematically conducted a series of temperature‐dependent emission spectra and time‐resolved decay measurements (Figure 3e,f and Figures S16,S17, Supporting Information). The results from these tests demonstrate a gradual decrease in both the intensity and lifetime of the MOHs as the temperature rises, affirming that the afterglow emission of MOHs falls into the category of ultralong RTP.^[^ ^16^ ^]^

**Figure 4:**
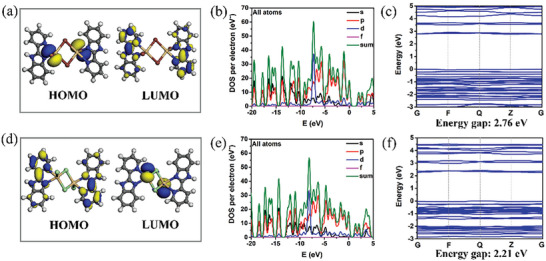
Calculated molecular orbitals, TDOS and PDOS, and band structures around Fermi energy levels for L‐CdCl_2_ (a–c) and L‐CdI_2_ (d–f).

To enhance our understanding of the photoluminescent mechanism of MOHs, electron‐density distributions, density of states (DOS), and band structures were calculated by using periodic density functional theory (DFT). Frontier orbital analyses revealed that the highest occupied molecular orbital (HOMO) and the lowest unoccupied molecular orbital (LUMO) are localized on halogen ions (Cl^−^ or I^−^) and organic ligands, respectively. This localization indicates the presence of halogen‐to‐ligand charge transfer (XLCT) processes (**Figure** **4**a,d). Furthermore, our analysis identified the occurrence of both MLCT and LE states in the MOHs by examining other relevant molecular orbitals (Figures S18,S19, Supporting Information). These findings confirm that the excitation‐dependent luminescence arises from the coexistence of multiple excited states.^[^ ^17^ ^]^ Total electronic density of states (TDOS) and partial electronic density of states (PDOS) investigations reveal that the valence bands (VB) primarily originate from the *p* orbitals of halogen ions, while the conduction bands (CB) are primarily composed of the *p* orbitals of C and N atoms (Figures S20,S21, Supporting Information). Therefore, halogen ions play an important role in the luminescence of MOHs. To explore the relationship between the structures and luminescent properties, we calculated DOS and band gap values as presented in Figure 4b,c,e,f. It is evident that the band gap of L‐CdCl_2_ (2.76 eV) exceeds that of L‐CdI_2_ (2.21 eV), indicating that the more electronegative Cl is better suited to stabilize the halide‐based VB, resulting in a blue shift in emission from the singlet excited state. This conclusion aligns reasonably well with experimental results (L‐CdCl_2_: 2.91 eV, 425 nm; L‐CdI_2_: 2.21 eV, 560 nm).

**Figure 5:**
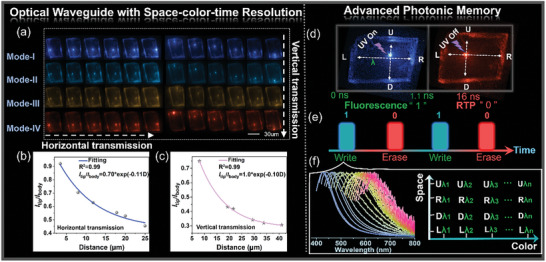
a) PL images obtained from individual L‐CdCl_2_ crystals by exciting different positions along horizontal and vertical directions in multiple excitation modes, respectively; Upon irradiation with 375 nm laser at room temperature, intensity ratio Itip/Ibody against the distance D along b) horizontal and c) vertical directions, respectively. d–f) Advanced photonic memory based on triple‐resolution optical waveguides.

Drawing inspiration from the remarkable color‐tunable luminescence and well‐defined microstructures, we conducted an in‐depth investigation of the photonic properties. Impressively, when subjected to unfocused UV light, the 2D microsheets of L‐CdCl_2_ and L‐CdI_2_ exhibited brighter blue and yellow light beams at their edges, indicative of typical 2D optical waveguide behaviors (Figures S22,S23, Supporting Information).^[^ ^6^, ^18^ ^]^ To further delve into the intriguing ex situ spatially‐resolved optical waveguide phenomenon, we selected individual 2D microcrystals of micron dimensions (45 µm in length and 35 µm in width for L‐CdCl_2_; 117 µm in length and 87 µm in width for L‐CdI_2_) for a detailed examination of their unique light propagation characteristics (Figure S24, Supporting Information). Detailed spatially resolved PL images were obtained under 375 nm laser excitation for L‐CdCl_2_ and 405 nm laser excitation for L‐CdI_2_ at various local positions on the 2D microcrystals (**Figures** **5**a and S25, Supporting Information). When the laser beam was precisely focused within the plane, the generated photon signals were confined and transmitted from the excitation point to all edges. The emission intensity remained constant at the excitation site, but gradually decreased at the edges as the propagation distance increased when the laser was moved both horizontally and vertically. To assess the 2D waveguide performance, we recorded the PL intensity at the excited site along the body of the 2D microcrystals (referred to as *I*_body_) and at the edge (referred to as *I*_tip_). Subsequently, we calculated the optical loss coefficient (*α*) using the single‐exponential formula: *I*_tip_/*I*_body_ = *A* exp (‐*αD*), where *A* represents the ratio of energy escaping and propagating, and *D* signifies the propagation distance between the emitting edge and the excited position along the horizontal or vertical direction.^[^ ^19^ ^]^ As shown in Figure 5b,c and Figure S26, Supporting Information, the *α*_horizontal_ and *α*_vertical_ are calculated as 0.10 and 0.11 dB µm^−1^ for L‐CdCl_2_, and as 0.062 and 0.063 dB µm^−1^ for L‐CdI_2_, respectively, thus revealing that the optical waveguide property is isotropic in L‐CdCl_2_ and L‐CdI_2_.^[^ ^20^ ^]^ Notably, the *α* values were found to be lower than those reported for molecule‐based optical waveguide materials, providing evidence of the excellent multichannel photon transmission capabilities of MOHs (Table S4, Supporting Information).

Inspired by the excitation‐dependent PL properties and time‐resolved RTP properties exhibited by MOHs, we embarked on an in‐depth study of individual microcrystals possessing spatially‐resolved optical waveguide characteristics. To do so, we detected and gathered emission signals under various excitation wavelengths in both prompt and delayed regions. As shown in Figure 5a, bright blue, cyan, and yellow coupled light beams can be observed at all edges of the L‐CdCl_2_ microcrystal when excited by 375, 405, and 488 nm beams in prompt mode (Mode‐I to Mode‐III), respectively. Moreover, the edges of the L‐CdCl_2_ microcrystal emitting orange light in delayed mode (375 nm excitation) can be defined as Mode‐IV (Table S5, Supporting Information). In summary, our experiments confirmed that these engineered MOH microcrystals can produce a wide range of tunable emission colors, which were also observed in L‐CdI_2_ microcrystals. As depicted in Figures S27,S28, Supporting Information, yellowish‐orange and orange emissions were observed at the edges of L‐CdI_2_ microcrystals under different excitation modes, respectively. Consequently, MOHs display multichannel 2D optical waveguide properties characterized by tunable full‐color emission and photon signal propagation pathways, which render MOH systems suitable for advanced photonic communication, significantly enhancing information transmission capacity and improving the information security level.

By leveraging the time‐space‐color 2D optical waveguide properties, we devised an all‐photonic memory system centered on the L‐CdCl_2_ microcrystals, enabling high‐density information writing and erasure operations. This photonic memory system operates on the foundation of time‐resolved characteristics. Specifically, when laser beams excite a point in the 2D optical waveguide, fluorescence could be detected in the 0–1.1 ns time range, which we term the “1” state. When the photon signal decays time is equal to 16 ns (termed as “0” state), the short‐lived fluorescence signal of the L‐CdCl_2_ will decay to 0 and only the orange‐red afterglow will be observed (Figure 5d). Therefore, we can successfully write into or erase stored information in the L‐CdCl_2_ microcrystal by switching the short‐lived fluorescence emission mode and long‐lived afterglow emission mode. Importantly, this system boasts high‐density information storage capacity, attributed to its space‐color resolution characteristics. As illustrated in Figure 5e, four‐photon signal transmission channels (U: Up; R: Right; D: Down; L: Left) are situated at the four edges of the 2D optical waveguide. By using different laser beam wavelengths to excite a point in the 2D optical waveguide, we generate photon signals with varying emission wavelength (*λ*) on the nanosecond timescale, transmitted through the four transmission channels (Figure 5f). This implies that the information storage capacity can be increased by at least 4*n* (where *n* = 1, 2, 3 ^…^) times compared to one‐dimensional (1D) optical waveguides.

Multiblock heterojunctions featuring in situ space‐resolved emission colors have aroused widespread research interest due to their potential applications in information security. However, the as‐reported heterojunctions are almost based on the static luminescent signals.^[^ ^9^, ^21^ ^]^ Consequently, designing heterojunctions with multi‐resolved emission characteristics has become highly desirable. Incorporating RTP emission into single‐crystal heterojunctions with static luminescent signals presents an effective strategy to address this challenge. Epitaxial growth is an effective method for preparing single‐crystal heterojunctions, which refers to the deposition of crystalline material on the well‐defined surface of a crystalline substrate, with the overlayer having the same crystalline orientation as the substrate. A small lattice mismatch between two crystals is the key factor for realizing epitaxial growth, which minimizes the interfacial energy of hybrid nanostructures.^[^ ^22^ ^]^

**Figure 6:**
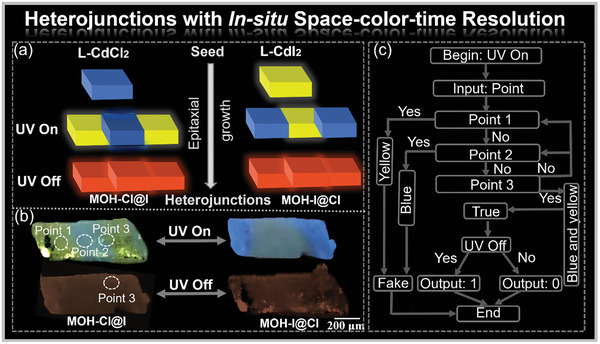
a) Schematic diagram of the epitaxial growth process; b) Photographs of heterojunctions MOHs‐Cl@I and MOHs‐I@Cl under the conditions of UV On and UV Off (*λ*_ex_ = 365 nm); c) Schematic diagram of the information encryption and decryption processes based on the heterojunction MOH‐Cl@I.

Herein, taking advantage of isostructural MOHs of L‐CdCl_2_ and L‐CdI_2_, we successfully fabricated multiblock heterojunctions (MOHs‐Cl@I and MOHs‐I@Cl) with in situ space‐time‐color resolution through an epitaxial growth process (**Figure** **6**a). For example, the MOHs‐Cl@I heterostructure exhibits blue emission from L‐CdCl_2_ in the central region and yellow emission from L‐CdI_2_ in the two end regions upon UV light (365 nm) irradiation (Figure 6b). Upon removal of the UV light, both MOHs‐Cl@I and MOHs‐I@Cl heterostructures emit orange light due to the similar RTP emission characteristics of both L‐CdCl_2_ and L‐CdI_2_. To better understand the formation of these multiblock heterojunctions, simulated morphology was performed on the L‐CdCl_2_ and L‐CdI_2_. Combined with the crystal plane parameters (Tables S6,S7, Supporting Information) and crystal growth direction (Figure S29, Supporting Information), it can be inferred that the heterogeneous interface between L‐CdCl_2_ and L‐CdI_2_ crystals is (0 1 −1) crystal plane. As shown in Tables S6,S7, Supporting Information, the d‐spacing values of the (0 1 −1) crystal plane are 7.16 and 7.29 Å for L‐CdCl_2_ and L‐CdI_2_ crystals, respectively. The lattice mismatching ratio of L‐CdCl_2_ and L‐CdI_2_ crystals at the junction region can be calculated as low as 1.7%,^[^ ^23^ ^]^ suggesting that the low lattice mismatch ratio facilitates the nucleation and epitaxial growth of L‐CdCl_2_ (or L‐CdI_2_) on the side facet of L‐CdI_2_ (L‐CdCl_2_) to achieve the single crystal heterostructure. As shown in Figure S30, Supporting Information, the characteristic diffractions of L‐CdCl_2_ and L‐CdI_2_ crystal at the (0 1 −1) plane have the same angle, which further confirms that the (0 1 −1) crystal plane is the heterogeneous interface.

By virtue of the in situ space‐time‐color resolved emission capabilities of the heterojunctions, we have designed a photonic logical gate utilizing the MOH‐Cl@I crystal as a microchip (Figure 6c). The edge of this microchip emitted distinct colors before and after exposure to UV irradiation, with the excited location serving as the input value. First, an input of Point 1, characterized by yellow emission, or Point 2, displaying blue emission, would immediately trigger a false message. Conversely, an input of Point 3, exhibiting both yellow and blue emission, would generate a true message. Subsequently, a photonic calculation system based on time‐resolved emissions was conducted at Point 3. Particularly, when Point 3 was illuminated with UV light, it concurrently emitted yellow and blue light, denoted as “1.” Upon deactivating the UV light, the dual emission transitioned into orange RTP emission, marked as “0.” This approach allowed us to harness the in situ space‐time‐color resolution characteristics capabilities inherent to the MOH‐Cl@I heterojunction, facilitating multiple applications in anti‐counterfeiting and information storage.

## 3 Conclusion

In summary, we have successfully produced novel isostructural 2D microcrystals of color‐tunable MOHs (L‐CdCl_2_ and L‐CdI_2_), which possess time‐resolved RTP properties. These microcrystals were easily fabricated through a straightforward solution self‐assembly process. Thanks to their highly ordered molecular stacking structures, both L‐CdCl_2_ and L‐CdI_2_ demonstrate the capability to transmit tunable photon signals through multiple channels, capitalizing on their ex situ space‐resolved 2D optical waveguide characteristics. This remarkable feature largely enhances photonic storage capacity. Of particular significance is our achievement in dynamically controlling in situ space‐resolved MOHs heterojunctions by integrating L‐CdCl_2_ and L‐CdI_2_ microcrystals through a stepwise epitaxial growth process. Therefore, this work not only represents the pioneering attempt to integrate full‐color 2D optical waveguide capabilities with heterojunction engineering in MOHs but also provides valuable guidance for the rational design of multi‐resolved luminescence systems, which may open up new avenues for all‐photonic logical computation.

## 4 Experimental Section

### Materials

All the reagents were purchased from the Sigma Chemistry Co. Ltd. and used without further purification. Distilled water was prepared in the lab.

### Preparation of Metal Halide Hybrid Crystals

Synthesis of L‐CdCl_2_: 2‐PyBIM (0.020 g, 0.10 mmol) and CdCl_2_^.^2.5H_2_O (0.045 g, 0.20 mmol) were dissolved in 4 mL of mixed solvent (MeCN: H_2_O = 3:1), the colorless crystals were obtained by heating to 95 °C for 72 h. Yield: ≈40% for L‐CdCl_2_ based on CdCl_2_.

### Synthesis of L‐CdI_2_

2‐PyBIM (0.020 g, 0.10 mmol) and CdI_2_ (0.073 g, 0.20 mmol) were dissolved in 4 mL of mixed solvent (MeCN: H_2_O = 3:1), the colorless crystals were obtained by heating to 95 °C for 72 h. Yield: ≈50% for L‐CdI_2_ based on CdI_2_.

### Synthesis of MOH‐Cl@I

1) 2‐PyBIM (0.020 g, 0.10 mmol) and CdCl_2_^.^2.5H_2_O (0.045 g, 0.20 mmol) were dissolved in 4 mL of mixed solvent (MeCN: H_2_O = 3:1), then sealed in a glass bottle (10 mL) and heated to 95 °C for 72 h, and the crystals were sucked out using a straw as seed. 2) Crystals obtained in the first step were directly added to the formamide solution, including CdI_2_ (0.073 g, 0.20 mmol) and 2‐PyBIM (0.020 g, 0.10 mmol), then sealed in a glass bottle (15 mL) and heated to 95 °C for 72 h, and the multiblock heterostructure crystals were prepared successfully.

### Synthesis of MOH‐I@Cl

The procedure is the same with MOH‐Cl@I.

### Characterizations

The single‐crystal X‐ray diffraction data of these samples were collected on a Rigaku XtalLAB Synergy diffractometer at 100 K with Cu‐K*α* radiation (*λ* = 1.54184 Å). SHELX‐2016 software was used to solve and refine the structure.^[^ ^24^ ^]^ FT‐IR spectra were recorded in the range of 4000–400 cm^−1^ on a Tensor 27 OPUS (Bruker) FT‐IR spectrometer. Solid UV–vis absorption spectra were carried out on a Shimadzu UV‐3600 spectrophotometer with BaSO_4_ as a standard. TGA tests were measured from room temperature to 800 K with a heating rate of 10 K min^−1^ on a Perkin–Elmer Diamond SII thermal analyzer under the N_2_ atmosphere. The relevant PL tests and time‐resolved lifetime for samples were measured on an FLS‐980 fluorescence spectrometer. The UV lamp used to take photos for fluorescence and afterglow photos was 2 W. PL microscope images of crystals were taken under the OLYMPUS IXTI fluorescence microscope. Photographs for the afterglow images were captured under iPhone SE. Spatially resolved PL images and spectra of the crystals were taken with the ISSQ2 FLIM/FFS confocal system (ISS Inc.). The system was attached to a Nikon inverted microscope, equipped with the Nikon 4X/0.2 NA objective lens. Diode lasers with 375, 405, and 488 nm were used for the excitation of the samples. The spot sizes were 1.2 µm (laser wavelength: 375 nm), 1.3 µm (laser wavelength: 405 nm), and 1.5 µm (laser wavelength: 488 nm), respectively. The angle between the incident laser and the horizontal plane of the optical waveguide was 90°. The beam could be transferred and calibrated through the single‐mode fiber (NA = 0.22). Polarization of the incident light: S/P = 100/1 (S: Senkrecht; P: Parallel). The images were acquired using a CMOS detector from TUCSON (model MI chrome 6) and MosaicV2.1 software. The scanning electron microscopy (SEM) images were taken on a field emission scanning electron microscopy (FESEM, Hitachi S‐8010).

### Theoretical Calculations

Electronic structure calculations were performed with the periodic DFT method by using the Dmol3 module in the Material Studio software package.^[^ ^25^ ^]^ The initial configurations were fully optimized by the Perdew–Wang (PW91) generalized gradient approximation (GGA) method with the double numerical basis sets plus polarization function (DNP).^[^ ^26^ ^]^

## Conflict of Interest

The authors declare no conflict of interest.

## Supporting information

Supporting Information

## Data Availability

The data that support the findings of this study are available in the supplementary material of this article.
