# Supplementary material for: Integrating Full‐Color 2D Optical Waveguide and Heterojunction Engineering in Halide Microsheets for Multichannel Photonic Logical Gates
Source: Adv Sci (Weinh). 2024 Feb 29;11(17):2310262. doi: 10.1002/advs.202310262 (PMC11077683; doi:10.1002/advs.202310262)
Supplement: Supplementary file 1 — Supporting Information [file ADVS-11-2310262-s001.pdf]

ADVANCED SCIENCE
Open Access

Supporting Information

for *Adv. Sci.*, DOI 10.1002/advs.202310262

Integrating Full-Color 2D Optical Waveguide and Heterojunction Engineering in Halide Microsheets for Multichannel Photonic Logical Gates

Chang Xing, Bo Zhou, Dongpeng Yan and Wei-Hai Fang*

Integrating Full-color 2D Optical Waveguide and Heterojunction Engineering in Halide Microsheets for Space-time-resolved Photonic Logical Gates

Chang Xing, Bo Zhou, Dongpeng Yan and Wei-Hai Fang*

Key Laboratory of Theoretical and Computational Photochemistry, Ministry of Education, College of Chemistry, Beijing Normal University, Beijing 100875, P. R. China

*Corresponding Author: yandp@bnu.edu.cn

Table of Contents

1. Materials and Methods

2. Figures (Figure S1 - Figure S30) and Tables (Table S1 - Table S7)

3. Supporting References

1. Materials and Methods

Materials

All the reagents (2-(2-pyridyl)benzimidazole, zinc chloride and hydrochloric acid) were purchased from the Sigma Chemistry Co. Ltd. and used without further purification. Distilled water was prepared in our lab.

Preparation of metal halide hybrid crystals

Synthesis of **L-$CdCl_2$**: 2-PyBIM (0.020 g, 0.10 mmol) and $CdCl_2 \cdot 2.5H_2O$ (0.045 g, 0.20 mmol) were dissolved in 4 mL mixed solvent (MeCN: H_2O = 3:1), the colorless crystals were obtained by heating to 95 °C for 72 hours. Yield: *ca.* 40% for L-$CdCl_2$ based on $CdCl_2$.

Synthesis of **$CdCl_2$**: 2-PyBIM (0.020 g, 0.10 mmol), CdI_2 (0.073 g, 0.20 mmol) were dissolved in 4 mL mixed solvent (MeCN: H_2O = 3:1), the colorless crystals were obtained by heating to 95 °C for 72 hours. Yield: *ca.* 40% for L-$CdCl_2$ based on CdI_2.

Yield: *ca.* 50% for L-CdI_2 based on CdI_2.

Synthesis of **MOH-Cl@I:** (1) 2-PyBIM (0.020 g, 0.10 mmol) and $CdCl_2 \cdot 2.5H_2O$ (0.045 g, 0.20 mmol) were dissolved in 4 mL mixed solvent (MeCN: H_2O = 3:1), then sealed in a glass bottle (10 mL) and heated to 95 °C for 72 hours, crystals were sucked out using a straw as seed. (2) crystals obtained in first step were directly added to the formamide solution including CdI_2 (0.073 g, 0.20 mmol) and 2-PyBIM (0.020 g, 0.10 mmol), then sealed in a glass bottle (15 mL) and heated to 95 °C for 72 hours, the multiblock heterostructure crystals were prepared successfully.

Synthesis of **MOH-I@Cl:** The procedure is the same with MOH-Cl@I.

Characterizations

The single-crystal X-ray diffraction data of these samples were collected on a Rigaku XtalLAB Synergy diffractometer at 100 K with Cu-Kα radiation (λ = 1.54184 Å). SHELX-2016 software was used to solve and refine the structure.[1] FT-IR spectra were recorded in the range of 4000-400 cm^{-1} on a Tensor 27 OPUS (Bruker) FT-IR spectrometer. Solid

UV-vis absorption spectra were carried out on a Shimadzu UV-3600 spectrophotometer with $BaSO_4$ as a standard. TGA tests were measured from room temperature to 800 K with a heating rate of 10 K min^{-1} on a Perkin-Elmer Diamond SII thermal analyzer under the N_2 atmosphere. The relevant photoluminescence (PL) tests and time-resolved lifetime for samples were measured on FLS-980 fluorescence spectrometer. The UV lamp used to take photos for fluorescence and afterglow photos is 2 W. PL microscope images of crystals were taken under OLYMPUS IXTI fluorescence microscope. Photographs for the afterglow images were captured under iphone SE. Spatially resolved PL images and spectra of the crystals were taken with the ISSQ2 FLIM/FFS confocal system (ISS Inc.). The system was attached to a Nikon inverted microscope, equipped with the Nikon 4X/0.2 NA objective lens. Diode lasers with 375, 405 and 488 nm was used for the excitation of the samples. The spot sizes are 1.2 μm (laser wavelength: 375 nm), 1.3 μm (laser wavelength: 405 nm), and 1.5 μm (laser wavelength: 488 nm), respectively. The angle between the incident laser and the horizontal plane of the optical waveguide is 90°. The beam could be transferred and calibrated through the single-mode fiber (NA = 0.22). Polarization of the incident light: S/P = 100/1 (S: Senkrecht; P: Parallel). The images were acquired using a CMOS detector from TUCSON (model MI chrome 6) and MosaicV2.1 software. The scanning electron microscopy (SEM) images were taken on a field emission scanning electron microscopy (FESEM, Hitachi S-8010).

Theoretical calculations

Electronic structure calculations were performed with the periodic density functional theory (DFT) method by using Dmol3 module in Material Studio software package.[2] The initial configurations were fully optimized by the Perdew-Wang (PW91) generalized gradient approximation (GGA) method with the double numerical basis sets plus polarization function (DNP).[3]

2. Figures (Figure S1 - Figure S31) and Tables (Table S1 - Table S7)

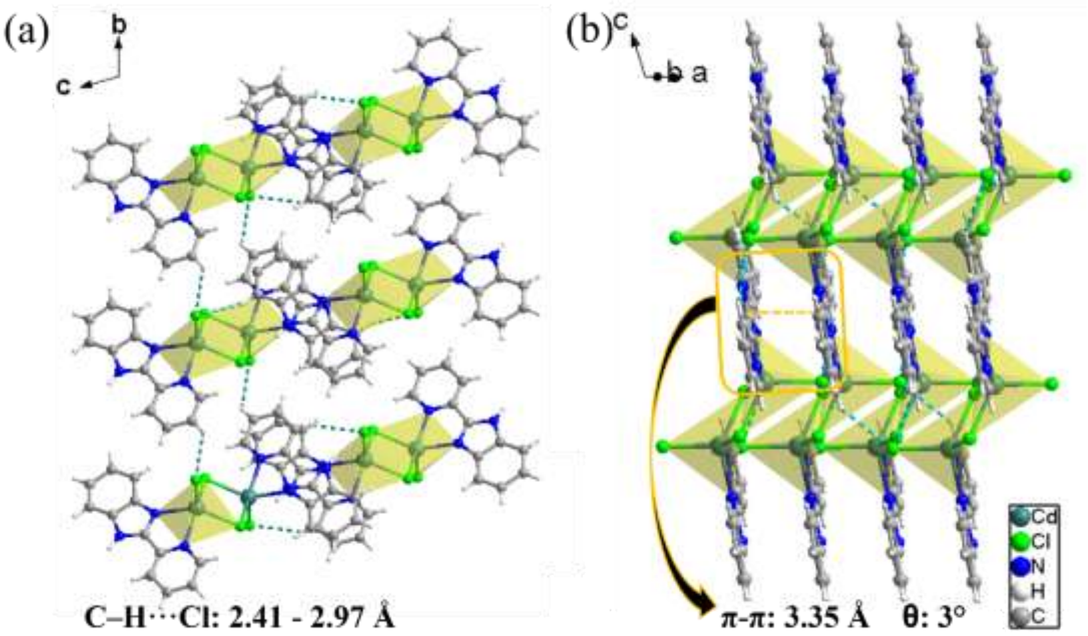

Figure S1. (a) and (b) Views of the packing of L-$CdCl_2$ projected based the hydrogen bonds (green dotted line) and π-π stacking (deep green dotted line) 3D structure.

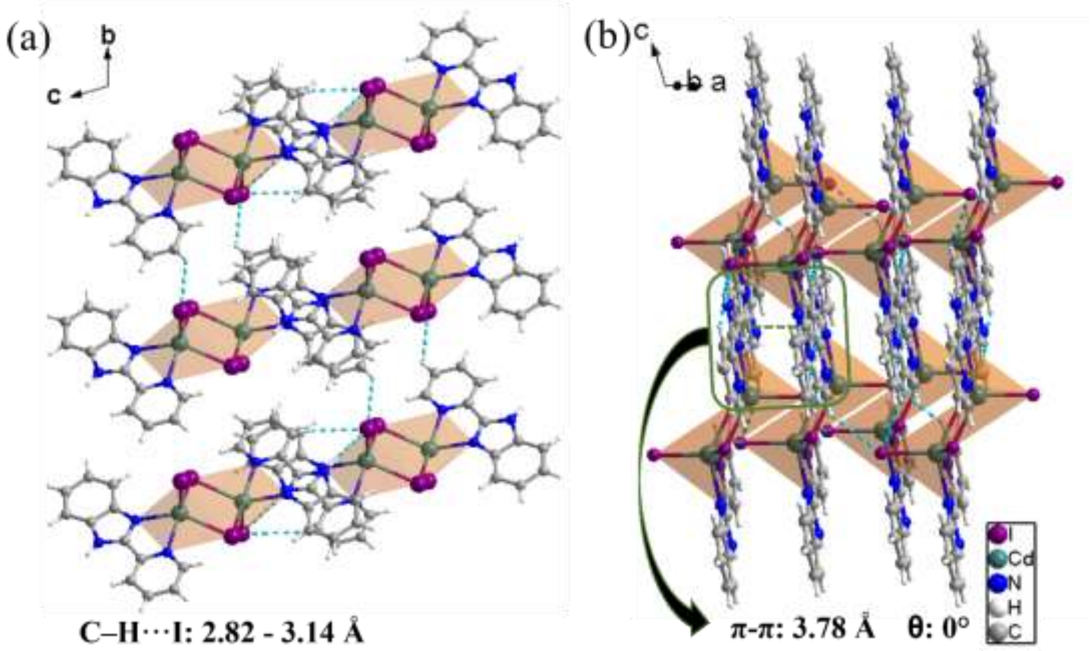

Figure S2. (a) and (b) Views of the packing of L-CdI_2 projected based the hydrogen bonds (green dotted line) and π-π stacking (deep green dotted line) 3D structure.

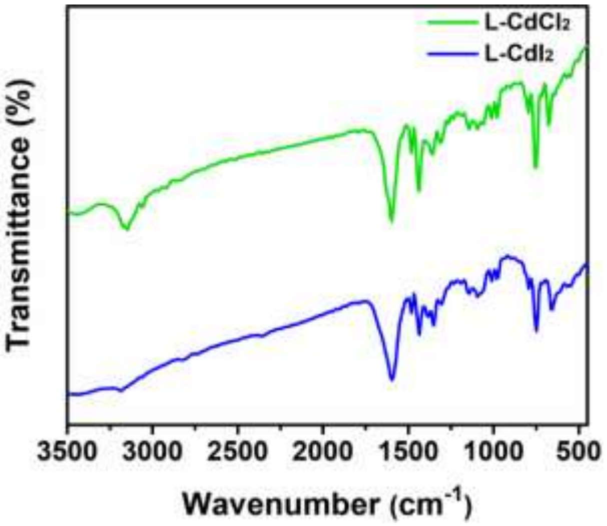

Figure S3. FT-IR spectra of MOHs.

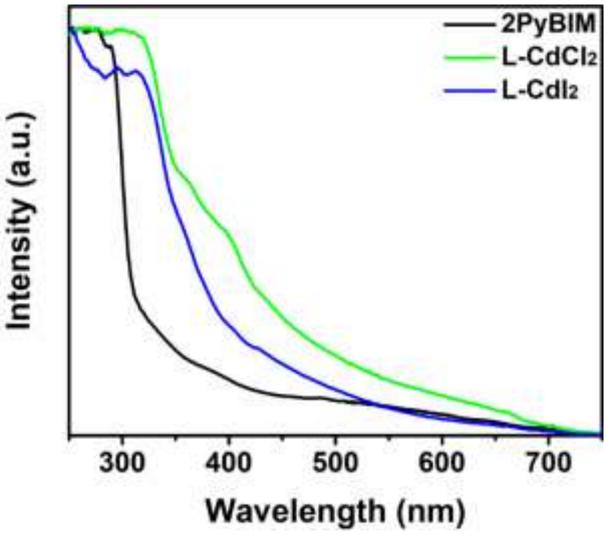

Figure S4. Solid-state UV-vis absorption spectra of organic ligand 2PyBIM, and metal-organic halide microcrystals of L-$CdCl_2$ and L-CdI_2 under ambient conditions.

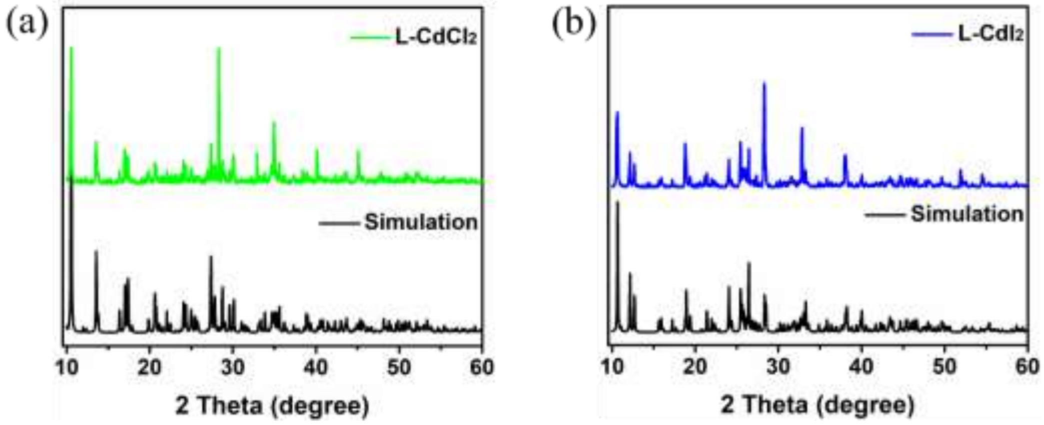

Figure S5. PXRD patterns for (a) L-$CdCl_2$ and (b) L-CdI_2.

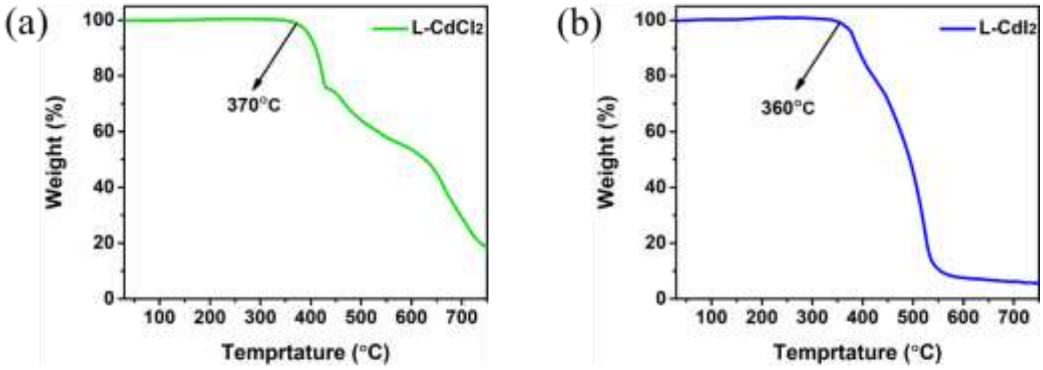

Figure S6. The TGA curves of (a) L-$CdCl_2$ and (b) L-CdI_2.

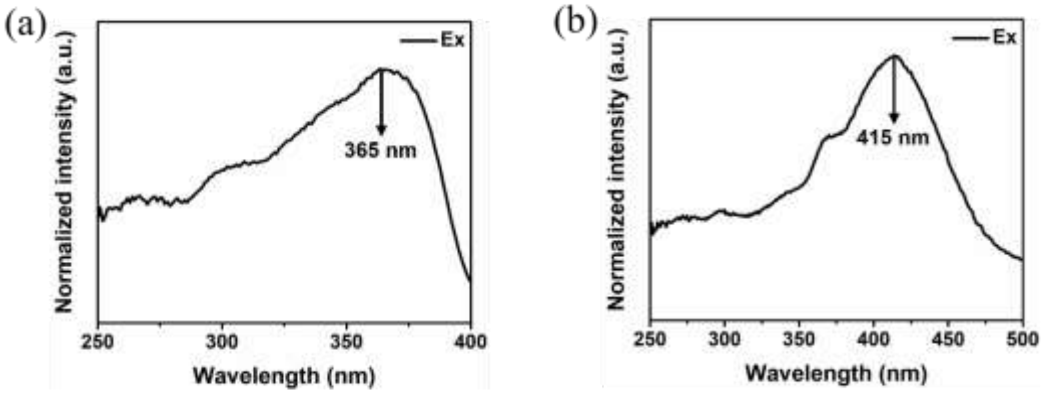

Figure S7. The prompt excitation spectra of (a) L-$CdCl_2$ and (b) L-CdI_2.

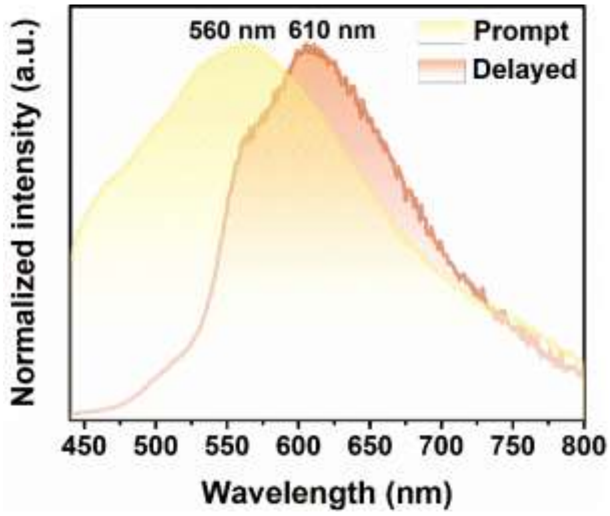

Figure S8. Prompt and delayed emission spectra of L-CdI_2 at room temperature.

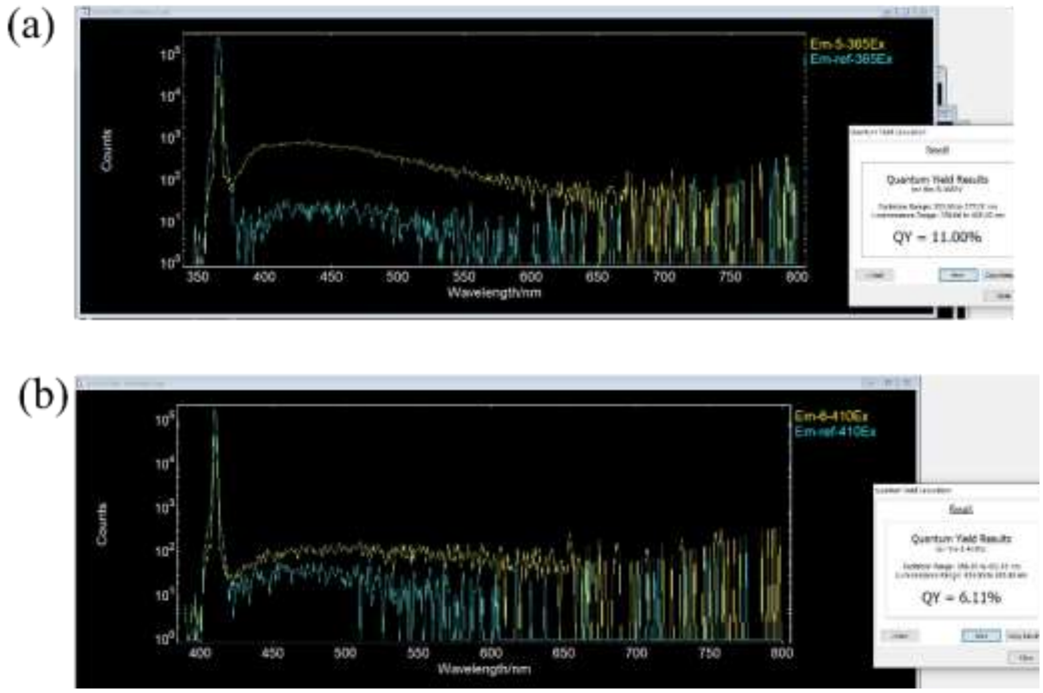

Figure S9. The detailed measurement profiles of PLQY for (a) L-$CdCl_2$ and (b) L-CdI_2 recorded in FLS980 fluorescence spectrometer.

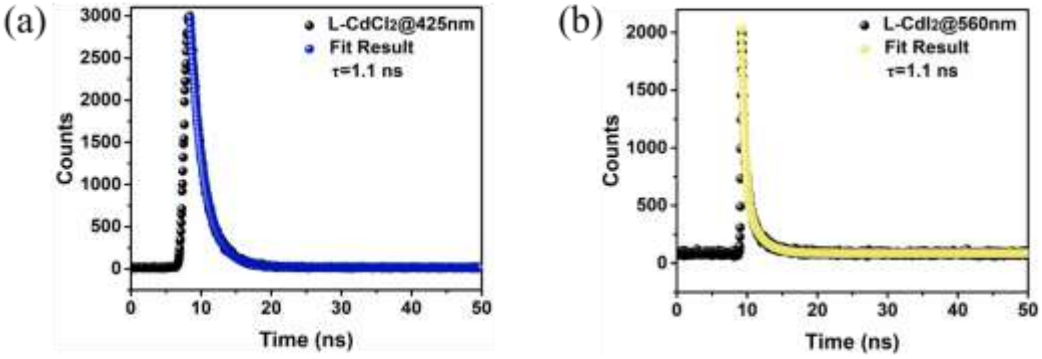

Figure S10. Time-resolved fluorescence prompt decay of (a) L-$CdCl_2$ and (b) L-CdI_2 at room temperature.

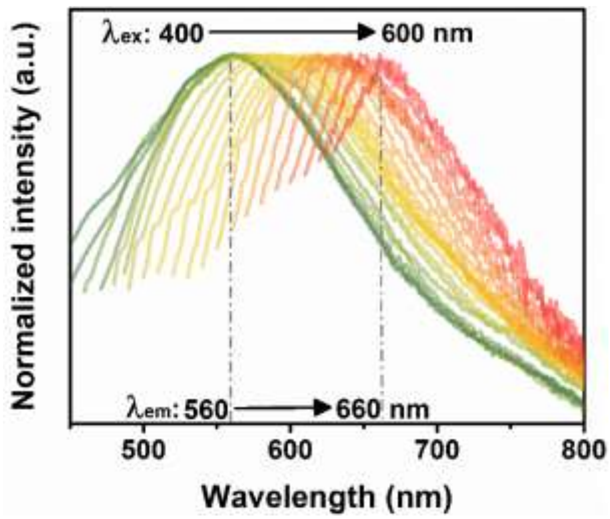

Figure S11. The excitation-dependent prompt emission spectra for L-CdI_2.

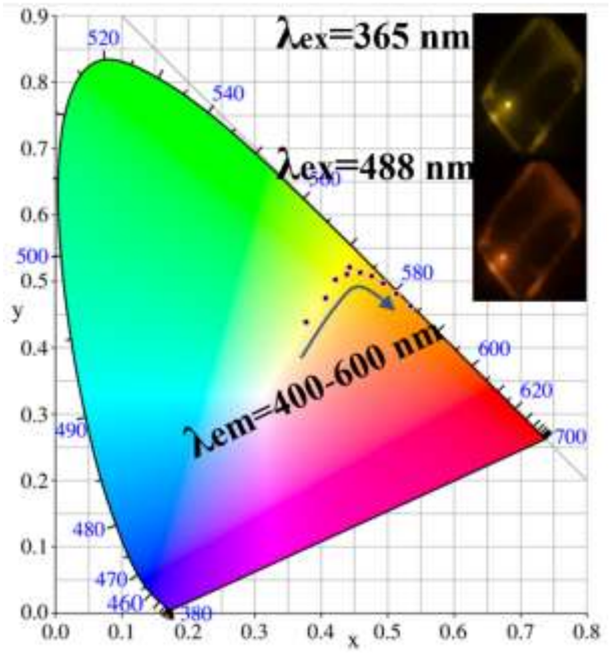

Figure S12. CIE coordinate diagram of L-CdI_2 as a function of the excitation wavelengths.

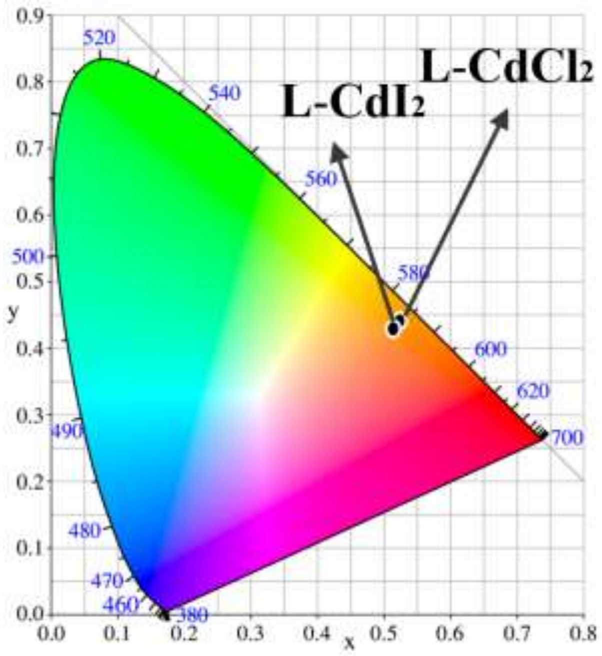

Figure S13. The chromaticity coordinates diagram for L-$CdCl_2$ and L-CdI_2 after ceasing the optimized wavelength, respectively.

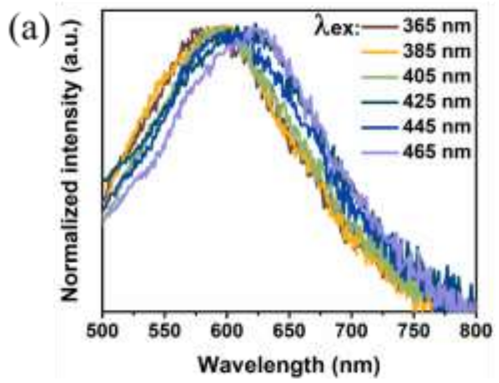

Figure S14. The delayed emission spectra of (a) L-$CdCl_2$ and (b) L-CdI_2 under the different excitation wavelengths.

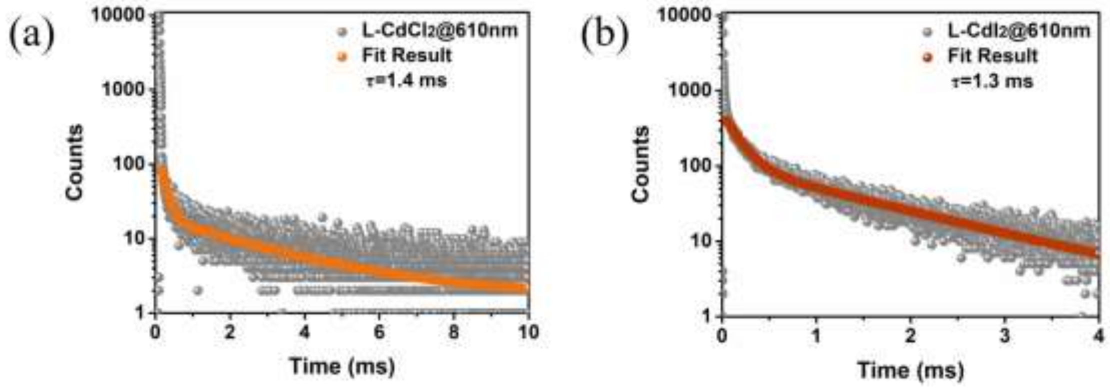

Figure S15. The time-resolved delayed decay of (a) L-$CdCl_2$ and (b) L-CdI_2 at room temperature.

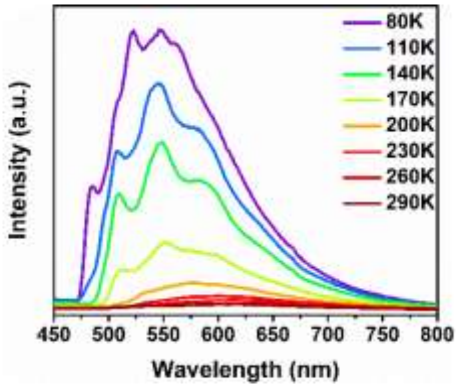

Figure S16. The delayed emission spectra of L-$CdCl_2$ under the different temperature.

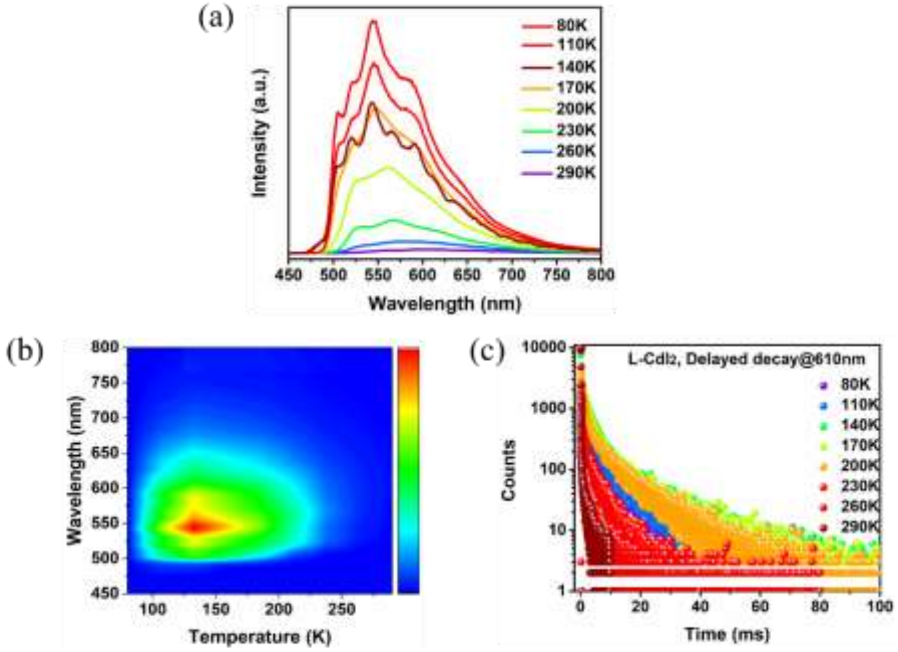

Figure S17. Temperature-dependent delayed spectra (a) and (b); time-resolved delayed decay at different temperatures ranging from 80 K to 290 K for L-CdI_2 (c).

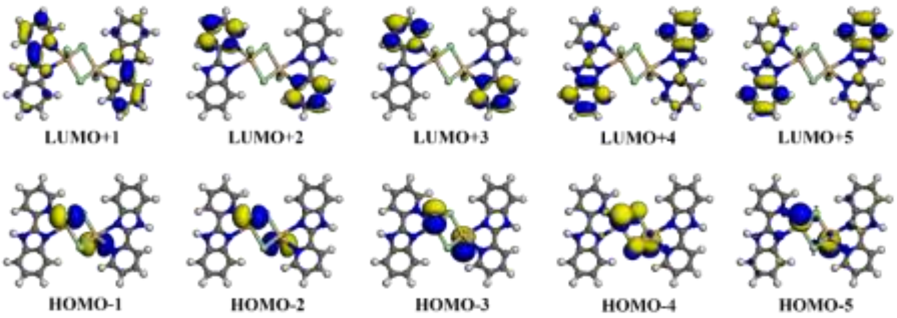

Figure S18. The other calculated molecular orbitals for L-$CdCl_2$.

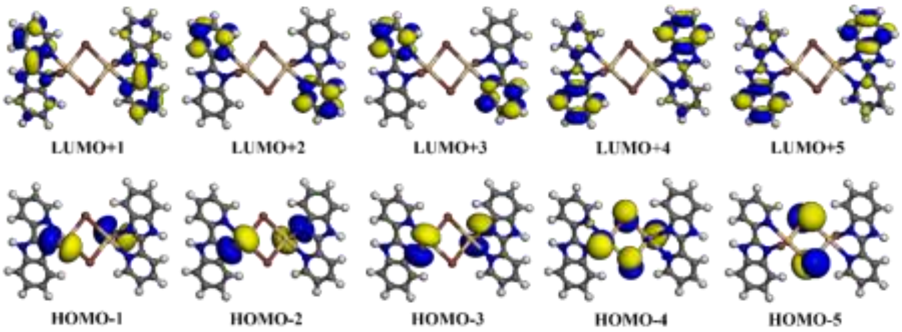

Figure S19. The other calculated molecular orbitals for L-CdI_2.

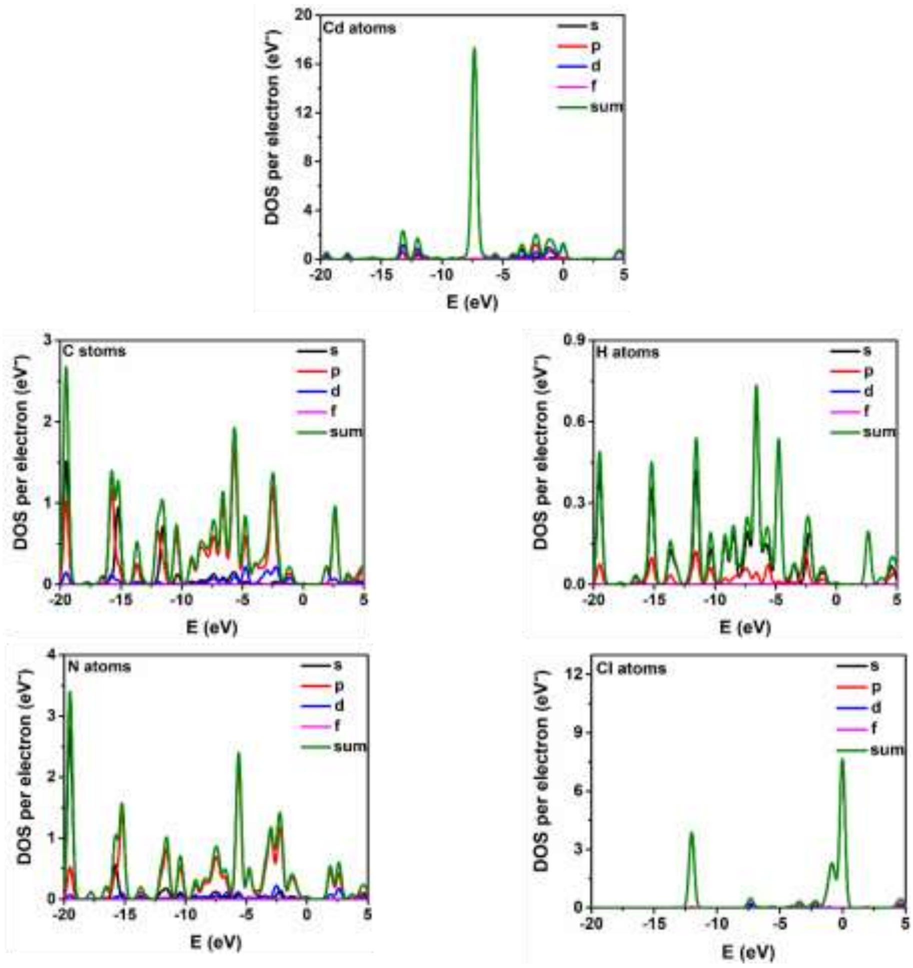

Figure S20. PDOS of the components in L-$CdCl_2$.

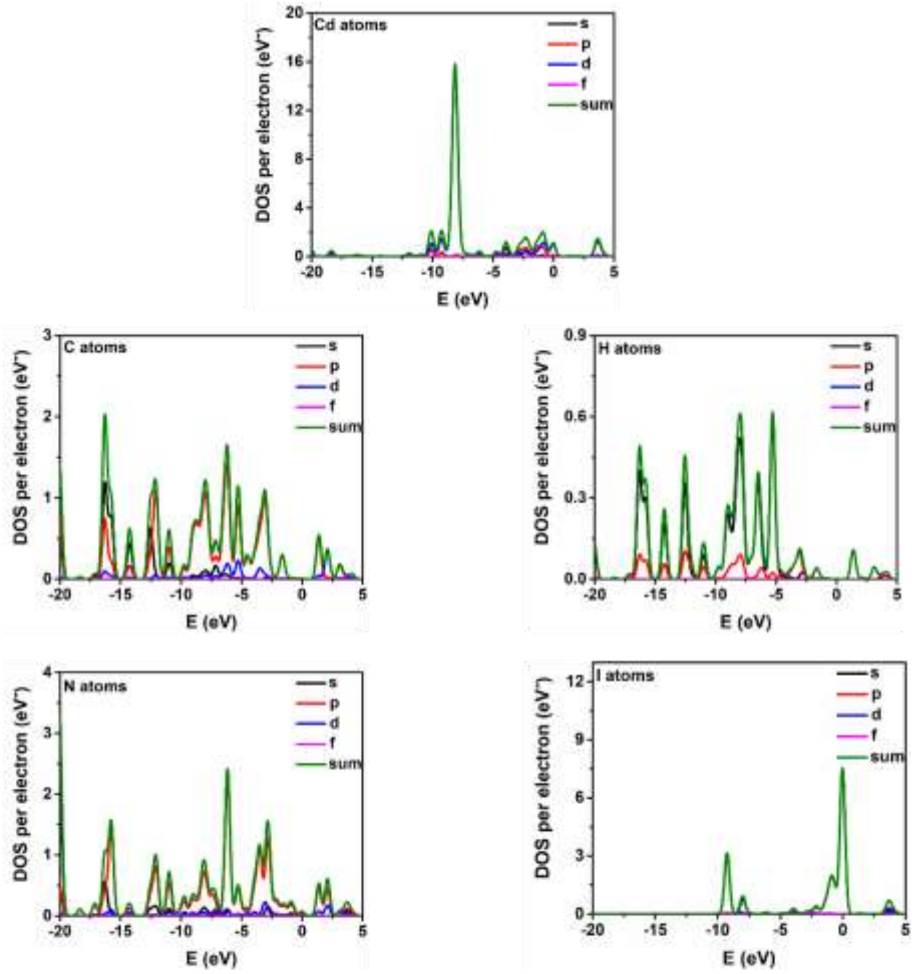

Figure S21. PDOS of the components in L-CdI_2.

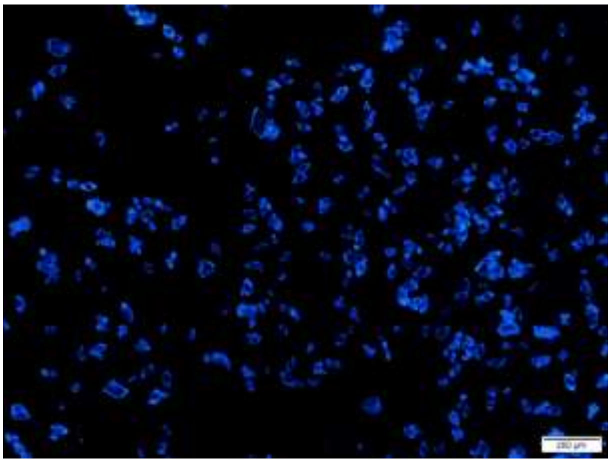

Figure S22. Fluorescence microscopy images of L-$CdCl_2$.

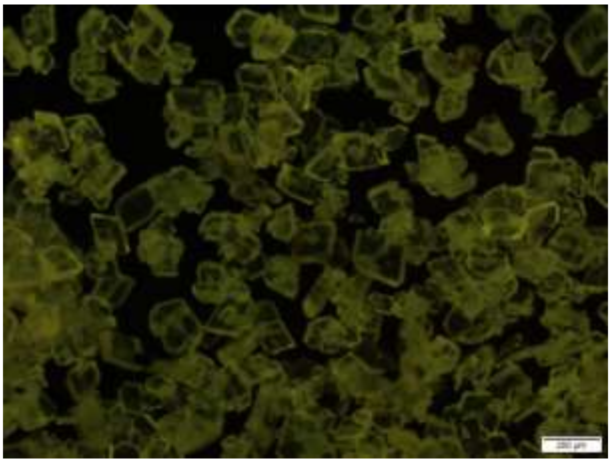

Figure S23. Fluorescence microscopy images of L-CdI_2.

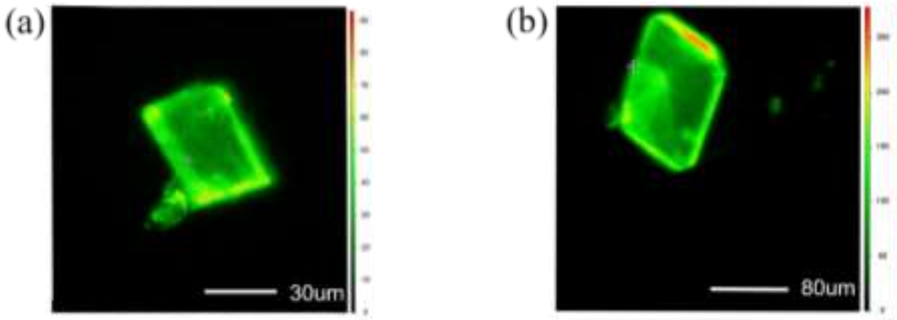

Figure S24. The size and morphology of (a) L-$CdCl_2$ and (b) L-CdI_2 were used for performing optical waveguide tests.

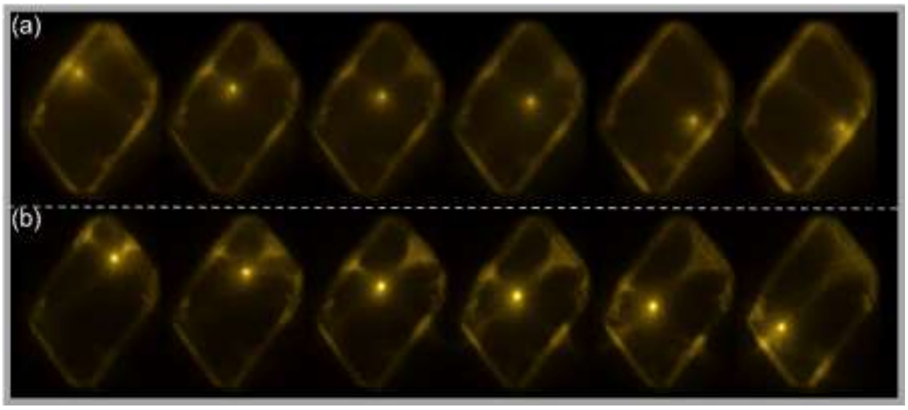

Figure S25. The prompt PL images obtained from an individual L-CdI_2 crystal by exciting with a laser beam (λ =405 nm) at different positions along (a) horizontal and (b) vertical direction, respectively.

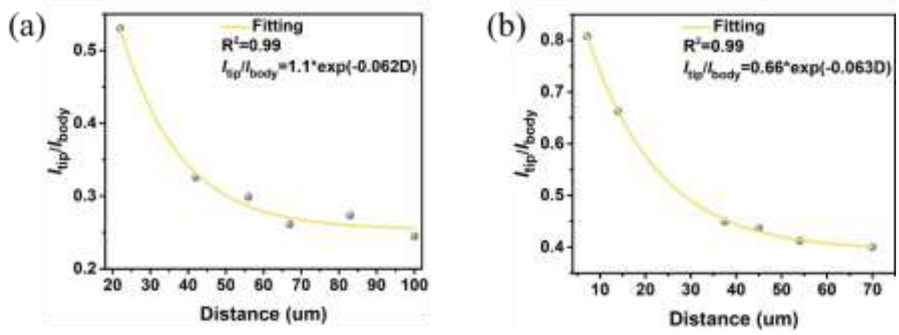

Figure S26.The optical loss coefficient for L-CdI_2. Intensity ratio I_{tip}/I_{body} against the distance D corresponding to Figure S23 along (a) horizontal and (b) vertical direction, respectively. Curves were fitted by the exponential decay function I_{tip}/I_{body} = A exp(-αD).

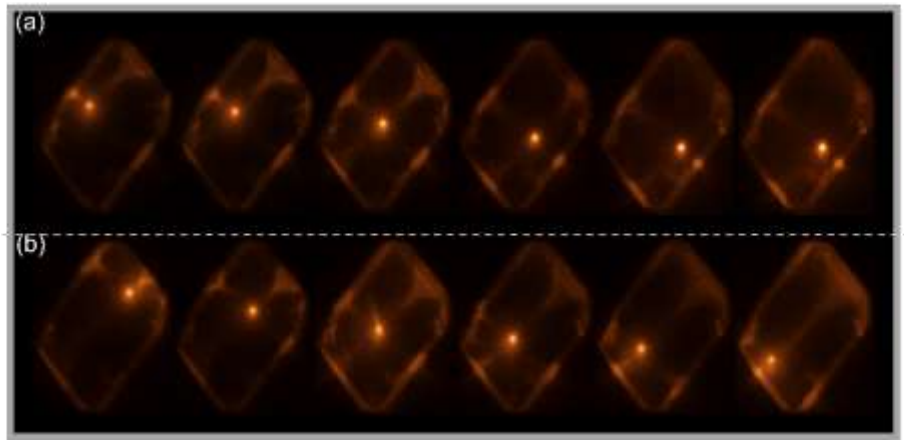

Figure S27. The prompt PL images obtained from an individual L-CdI_2 crystal by exciting with a laser beam (λ=488 nm) at different positions along (a) horizontal and (b) vertical direction, respectively.

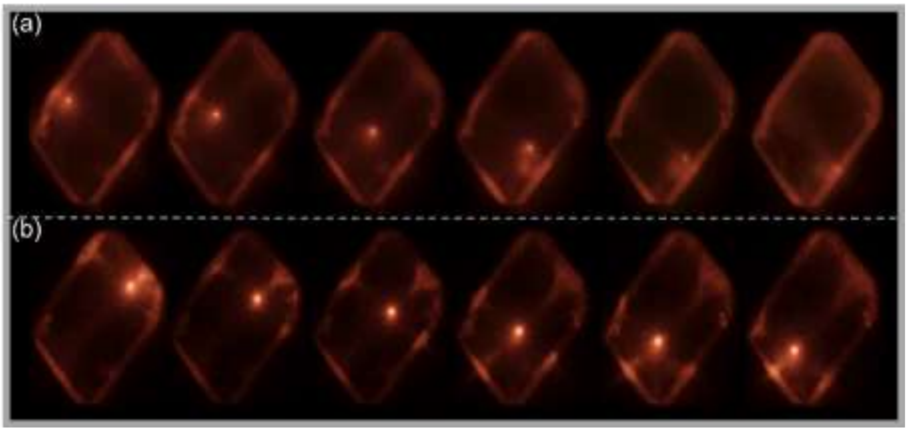

Figure S28. The delayed PL images obtained from an individual L-CdI_2 crystal by exciting with a laser beam (λ=405 nm) at different positions along (a) horizontal and (b) vertical direction, respectively.

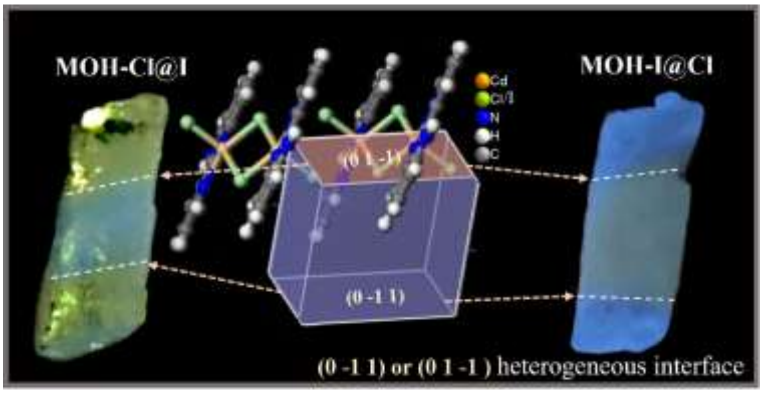

Figure S29. Predicted morphology and corresponding crystal faces for isostructural MOHs.

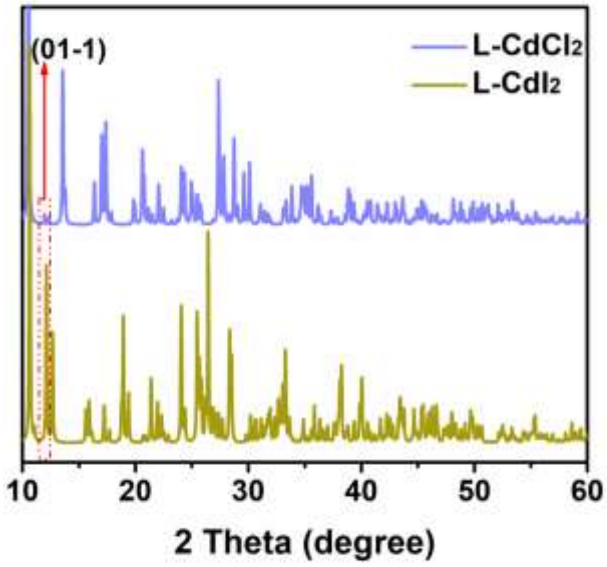

Figure S30. The XRD patterns of L-$CdCl_2$ and L-CdI_2.

Table S1. Crystallographic data for L-$CdCl_2$ and L-CdI_2 at 100 K.

	L-$CdCl_2$	L-CdI_2
**Molecular Formula**	$C_{24}H_{18}Cd_2Cl_4N_6$	$C_{24}H_{18}Cd_2I_4N_6$
**Molecular Weight**	635.66	1122.86
**Space group**	*P-1*	*P-1*
**Crystal system**	triclinic system	triclinic system
**a (Å)**	7.7782(2)	8.5596(5)
**b (Å)**	8.9030(2)	8.9715(6)
**c (Å)**	9.41761(16)	9.9484(8)
**α (deg)**	103.1051(19)	102.164(6)
**β (deg)**	100.782(2)	99.666(5)
**γ (deg)**	101.554(2)	105.969(5)
**V ($Å^3$)**	603.55(3)	696.95(9)
**Z**	1	1
**GooF**	1.126	1.016
**D (g cm^{-3})**	2.083	1.802
**μ (mm^{-1})**	18.419	5.981
**R_1 [I>2σ(I)]**	0.0300	0.0306
**wR_2 [I>2σ(I)]**	0.0815	0.0638

**CCDC Number**	2224204	2224205

Table S2. The distance parameter in L-$CdCl_2$ and L-CdI_2.

	**L-$CdCl_2$**	**L-CdI_2**
**C-H···Cl**	2.41 - 2.97 Å	-
**C-H···I**	-	2.82 - 3.14 Å
**Cd-Cl**	2.47 -2.58 Å	-
**Cd-I**	-	2.77 - 2.92 Å
**Cd-N**	2.28 - 2.34 Å	2.30 - 2.36 Å
**π–π**	3.35 Å	3.28 Å

Table S3. Phosphorescence lifetime <τ> of metal organic halide hybrids materials at room temperature.

	**L-$CdCl_2$**	**L-CdI_2**
**τ_1 (ms)**	0.016	0.163
**A_1 (%)**	35.4	27.0
**τ_2 (ms)**	4.598	1.394
**A_2 (%)**	64.6	73.0
**<τ> (ms)**	1.4	1.3
**χ^2**	1.2	1.0

The average lifetimes <τ> for the samples can be fitted by the function as follows:

$\tau_{ave} = \frac{A_1\,\tau_1^2 + A_2\,\tau_2^2}{A_1\,\tau_1 + A_2\,\tau_2}$, τ_1 and τ_2 are the decay times for the exponential components, and τ_{ave} is the average decay time calculated from τ_1 and τ_2, A_1 and A_2.

Table S4. Optical waveguide materials based on metal organic halide hybrids have been reported in recent years.

Sample name	OLC/dB μm^{-1}
L-$CdCl_2$ (this work)	0.10
L-CdI_2 (this work)	0.06
cis-o-BCB[4]	0.245
m-MSB[5]	0.276
AMN[6]	0.457
BT2O3Cz[7]	0.3260
DBBZL[8]	0.285
MW[9]	0.0995

Table S5. Excitation states in different modes.

Mode	Excitation wavelength (nm)	Delayed time (ms)
**Mode-I**	365	0
**Mode-II**	405	0
**Mode-III**	488	0
**Mode-IV**	365	1

Table S6. Crystal plane parameters for L-$CdCl_2$.

hkl	d_{hkl} (Å)
(0 0 -1)	8.8959
(0 1 0)	8.3874
(1 0 0)	7.3909
(0 1 -1)	7.1625
(1 0 -1)	6.5189
(1 -1 0)	6.4102
(1 -1 1)	5.2044

Table S7. Crystal plane parameters for L-CdI_2.

hkl	d_{hkl} (Å)
(0 0 1)	9.4399
(0 1 0)	8.3023
(1 0 0)	7.9880
(0 1 -1)	7.2909
(1 -1 0)	6.9906
(1 0 -1)	6.9810

(1 -1 1)	5.6667

3. References

[1] G. M. Sheldrick, *Acta Crystallogr., Sect. C: Struct. Chem.* **2015**, *71*, 3.

[2] a) F. Bouyera, G. Picard, Legendreb. J. Theochem. **1995,** *330,* 217; b) B. Delley, *J. Chem. Phys.* **2000**, *113*, 7756.

[3] J. P. Perdew, J. A. Chevary, S. H. Vosko, K. A. Jackson, M. R. Pederson, D. J. Singh, C. Fiolhais, *Phys. Rev. B* **1992**, *46*, 6671.

[4] Z. Z. Li, J. J. Wu, X. D. Wang, K. L. Wang, S. Zhang, W. F. Xie, L. S. Liao, *Adv. Optical Mater.* **2019**, *7,* 1900373.

[5] C.-F. Xu, Y. Yu, Q. Lv, C.-C. Yan, X.-D. Wang, L.-S. Liao, *Chinese Chem. Lett.* **2022**, *33*, 3255-3258.

[6] D. Tian, Y. Chen, *Adv. Optical. Mater.* **2021**, *9,* 2002264.

[7] D. Barman, M. Annadhasan, R. Chandrasekar, P. K. Iyer, *Chem. Sci.* **2022**, *13*, 9004-9015.

[8] H. Liu, Z. Bian, Q. Cheng, L. Lan, Y. Wang, H. Zhang, *Chem. Sci.* **2019**, *10*, 227-232.

[9] Z. Feng, T. Hai, Y. Liang, Q. Zhang, Y. Lei, *Angew. Chem. Int. Ed.* **2021**, *60*, 27046-27052.
